# Biomedical doctoral students’ research practices when facing dilemmas: two vignette-based randomized control trials

**DOI:** 10.1038/s41598-023-42121-1

**Published:** 2023-09-29

**Authors:** V.T Nguyen, M. K. Sharp, C. Superchi, G. Baron, K. Glonti, D. Blanco, M. Olsen, T.T Vo Tat, C. Olarte Parra, A. Névéol, D. Hren, P. Ravaud, I. Boutron

**Affiliations:** 1 Centre for Research in Epidemiology and Statistics (CRESS), Université Paris Cité and Université Sorbonne Paris Nord, Inserm, INRAE, 75004 Paris, France; 2 Department of Health Services Research, Institute of Population Health Sciences, University of Liverpool, Liverpool, UK; 3 Department of Psychology, Faculty of Humanities and Social Sciences, University of Split, Split, Croatia; 4 Royal College of Surgeons in Ireland University of Medicine and Health Sciences, Dublin, Ireland; 5 Statistics and Operations Research Department, Barcelona-Tech, UPC, Barcelona, Spain; 6 Department of Physiotherapy, Universitat Internacional de Catalunya, Barcelona, Spain; 7 Centre d’Epidémiologie Clinique, AP-HP, Hôpital Hôtel Dieu, 75004 Paris, France; 8 Academic Medical Center, University of Amsterdam, Amsterdam, The Netherlands; 9 Department of Statistics and Data Science, The Wharton School, University of Pennsylvania, Philadelphia, USA; 10 Department of Applied Mathematics, Computer Science and Statistics, Ghent University, Ghent, Belgium; 11 LIMSI, CNRS, Orsay, France; 12 Centre d’Épidémiologie Clinique, Hôpital Hôtel Dieu, 1 place du Parvis Notre-Dame, Cedex 4, 75089 Paris, France

**Keywords:** Medical research, Translational research

## Abstract

Our aim was to describe the research practices of doctoral students facing a dilemma to research integrity and to assess the impact of inappropriate research environments, i.e. exposure to (a) a post-doctoral researcher who committed a Detrimental Research Practice (DRP) in a similar situation and (b) a supervisor who did not oppose the DRP. We conducted two 2-arm, parallel-group randomized controlled trials. We created 10 vignettes describing a realistic dilemma with two alternative courses of action (good practice versus DRP). 630 PhD students were randomized through an online system to a vignette (a) with (n = 151) or without (n = 164) exposure to a post-doctoral researcher; (b) with (n = 155) or without (n = 160) exposure to a supervisor. The primary outcome was a score from − 5 to + 5, where positive scores indicated the choice of DRP and negative scores indicated good practice. Overall, 37% of unexposed participants chose to commit DRP with important variation across vignettes (minimum 10%; maximum 66%). The mean difference [95%CI] was 0.17 [− 0.65 to 0.99;], p = 0.65 when exposed to the post-doctoral researcher, and 0.79 [− 0.38; 1.94], p = 0.16, when exposed to the supervisor. In conclusion, we did not find evidence of an impact of postdoctoral researchers and supervisors on student research practices.

**Trial registration:** NCT04263805, NCT04263506 (registration date 11 February 2020).

## Introduction

Responsible and ethical conduct in biomedical research is essential for providing a strong evidence base to inform clinical care and health policies. In the biomedical sciences, major scientific misconduct has devastating consequences. For example, an infamous study that falsely linked autism to the triple vaccine for measles, mumps, and rubella (MMR)^1^ spawned a resurgence in the anti-vaccination movement, which snowballed in the following decades. Fraudulent clinical trials with cancer patients^2^ and fabricated results in stem cell research^3^ violate patients’ time and trust and create research waste. In recent years, a significant amount of research has been retracted due to misconduct. A study that reviewed 2047 retracted biomedical and life science research articles indexed by PubMed in detail found that 67.4% of retractions were attributable to scientific misconduct^4^. A recent investigation of COVID-19-related articles found many retractions due to issues with ethical approval, data integrity, study conduct, analysis, and reporting^5^. Although some researchers may view scientific misconduct as purposeful deceit, Nath et al. found that retractions in the biomedical literature were more likely to result from unintentional mistakes rather than from scientific misconduct^6^.

These instances are universally accepted as misconduct. Nevertheless, recent research suggests that scientists perceive many scenarios as ethical “grey areas,” rather than clearly wrong or right^7^. These “grey areas” were considered as questionable research practices and have been recently classified as detrimental research practices. The 1992 “*Responsible Science*” report published by the National Academies of Sciences, Engineering, and Medicine defined research misconduct as “*fabrication, falsification, or plagiarism in proposing, performing, or reporting research*”^8^. They were defined as a set of actions “*that violate traditional values of the research enterprise and that may be detrimental to the research process*,” but for which “*there is at present neither broad agreement as to the seriousness of these actions nor any consensus on standards for behavior in such matters*”. However, the 2017 ‘Fostering Integrity in Research’ report of the National Academies of Sciences, Engineering, and Medicine stated that “*many of the practices that up to now have been considered questionable research practices, as well as damaging behaviors by research institutions, sponsors, or journals, should be considered detrimental research practices (DRPs). Researchers, research institutions, research sponsors, journals, and societies should discourage and in some cases take corrective actions in response to DRPs*”^9^. These practices are prevalent in medical research and deteriorate research quality and credibility^10^.

Some attempts have been made to clarify this definition in further detail, providing a clear distinction between DRPs and misconduct. For example, Rezaeian et al. considered data fabrication, data falsification, plagiarism, and failing to obtain ethical approval as fraud or serious research misconduct^11^. According to the National Academies of Sciences, Engineering, and Medicine 2017 report, DRP could refer to selective reporting, guest authorship, retaining research materials, using inappropriate statistical or other methods of measurement to enhance the significance of research findings, and misrepresenting research results^9,11^.

Several research papers have assessed the prevalence of DRPs in different populations, but largely with experienced researchers^12–16^. In the field of psychology, an Italian study found that approximately 50% of researchers failed to report all dependent variables, and 39% of participants self-reported that they excluded data after an analysis to change the results^13^. Similar figures were also reported in the US^15^. In a survey of researchers in brain simulation research, 41% of respondents reported knowing researchers who selectively reported research outcomes, and 25% of respondents admitted to personally adjusting an analysis^17^.

Due to the pervasiveness and variability in the perceived seriousness of DRPs, early career researchers might find it difficult to avoid detrimental choices. PhD students are in vulnerable positions; they need publications to facilitate progress and are dependent upon their hosting team and supervisors. Mumford et al. described how researchers’ ethical decision-making can be influenced by the research climate and organizational norms^18^. Recent research on the prevalence of DRPs in doctoral theses showed that established researchers, who serve as PhD supervisors, play an influential role in students’ practices^19^. Situations such as inadequate mentoring or a supervisor’s lack of opposition to DRPs can encourage researchers to engage in questionable practices. This dynamic can heighten the potential occurrence of a DRP; however, there is little research exploring PhD students’ research practices.

Our aim was (1) to describe the research practices of biomedical PhD students facing dilemma situations related to research integrity and (2) evaluate the impact of inappropriate research environment on PhD students’ research practice. We particularly explored the impact of exposure to (a) a post-doctoral researcher who committed DRP in a similar situation and (b) the supervisor who did not oppose the DRP. Our hypothesis was that an inappropriate research environment could increase DRP performed by PhD students.

## Methods

We conducted two vignette–based randomized trials embedded in an online survey (NCT04263805 and NCT04263506, registration date 11 February 2020). A vignette can be defined as a brief story or scenario in which participants are asked a set of questions to examine their research practices. In this study, the vignettes were scenarios of research dilemmas that could result in DRPs. We defined DRP as “research practices other than Fabrication, Falsification, and Plagiarism (FFP) that are clearly detrimental to the research process”^9^.

This project was funded as part of the Marie Sklodowska–Curie European Joint Doctoral Training Program on Methods in Research on Research (MiRoR—http://miror-ejd.eu/). This study was approved by the Institutional Review Board of the Inserm (Comité d’Évaluation Éthique, IRB 00003888, Reference Number: 19-587). The study did not collect any identifying information from participants and was conducted in accordance with guidelines and regulations. The protocol is available at https://doi.org/10.5281/zenodo.7387752.

### Vignette development

First, we conducted a literature review to identify and classify different types and examples of dilemmas that PhD students may face and could result in DRPs. Two authors (DB, MO) searched PubMed on March 15, 2018, using “Questionable Research Practices[tw]” (including forward citations), yielding 54 articles. An additional 15 were obtained from consultation with experts within the Methods in Research on Research (MiRoR) consortium. Five authors (LB, DB, MO, MG, and MKS) extracted data from the sources using a standardized data extraction form, and 286 DRPs were identified. These were then classified by two researchers (DB and MO) according to the research phase in which they may occur: (i) hypothesis and study design, (ii) conduct (data collection/handling), (iii) analysis, (iv) reporting, (v) publication (e.g. authorship issues), and (vi) others (e.g. policies, ethics, and supervision/training). Disagreements were resolved through discussion with a senior researcher (IB).

To supplement the literature review, thirteen doctoral students from the MiRoR project conducted semi-structured interviews with colleagues after undergoing training in qualitative interviewing (http://miror-ejd.eu/second-training-event/). Doctoral students were located in six countries in Europe: Belgium, Croatia, France, the Netherlands, Spain, and the United Kingdom, and eligible participants (i.e. biomedical PhD students) were identified for interview from professional networks within their current country of residence. Participants engaged in semi-structured interviews either face-to-face, by phone, or via videoconferencing according to the circumstances and preferences of the interviewees. A semi-structured interview guide was developed by two researchers (VN and KG) with guidance from a senior researcher (DH) to support the interview process. Interviews were conducted in English between July and September 2017. All participants provided informed consent prior to the interviews and provided audio recordings. There were no restrictions on age, gender, year of PhD study, or level of research experience. The interview guide covered three main sections (Appendix 1).

(i) Interviewees’ academic background and level of research experience,
(ii) Interviewees’ perception of DRPs which is reflected by how they define DRPs,
(iii) Interviewees’ experiences with DRPs in their research environment and personal experiences of DRPs in their own research.

The interviews were transcribed verbatim and thematically analyzed using NVivo V.11.^20^, following the method outlined by Braun and Clarke^21^. All transcripts were coded independently by the researchers who conducted interviews. Based on the initial codes, two researchers (VN and KG) developed a coding scheme with themes and subthemes. After consulting senior researchers (IB and DH) who read a random sample of three transcripts to ensure that the developed themes fit the data, the codebook was refined, and a final version was developed.

Through discussions within the authorship team and consultation with experts from the MiRoR Consortium, ten DRPs of interest were selected that were (i) relevant to all biomedical disciplines and (ii) common to PhD students in biomedical research. Three researchers (VN, CS, TVT) collaboratively developed a case vignette for each of the 10 DRPs (Table 1). For each case vignette, the researchers created a dilemma in which the main character was a PhD student. The dilemmas concerned different research phases (e.g. conducting, reporting, and publication), each of which could result in a DRP. Three senior researchers (IB, DH, and AN) reviewed and revised the case vignette. To increase participants’ engagement with the content, the vignette was expressed as a short comic strip summarizing the situation, and the survey was translated into five different languages (English, French, German, Italian, and Spanish) by native speakers of the authorship team.

**Table 1:** Ten DRP selected for vignette development.

Detrimental research practice	Definition
Honorary authorship	Adding an author who doesn’t qualify for authorship (i.e. does not meet established criteria such as those put forth by the International Committee of Medical Journal Editors^21^)
Changing the focus and/or outcome of the study	Reporting statistically significant secondary outcomes as if they were primary outcomes
Exaggerating the importance of study results	Reporting conclusions that exaggerate the study findings
Hypothesizing after the results are known	Reporting data-driven hypotheses without disclosure to make results appear more significant
Peer review pressure	Following a peer reviewer’s request to modify study outcomes or conclusions to increase the possibility of acceptance by the journal
Salami slicing	Publishing the same data or results in two or more publications without full disclosure
Not reporting limitations	Failing to specify all limitations in sampling, data collection, and analysis
Fishing results	Running multiple analyses with similar procedures and only reporting those yielding significant results
Excluding data from analysis	Excluding participants only to reach the level of significance (e.g. through insufficiently justified outlier analyses)
Committing mistakes in analysis	Lacking intention of correcting mistakes in published research

### Trial design

**Figure 1:**
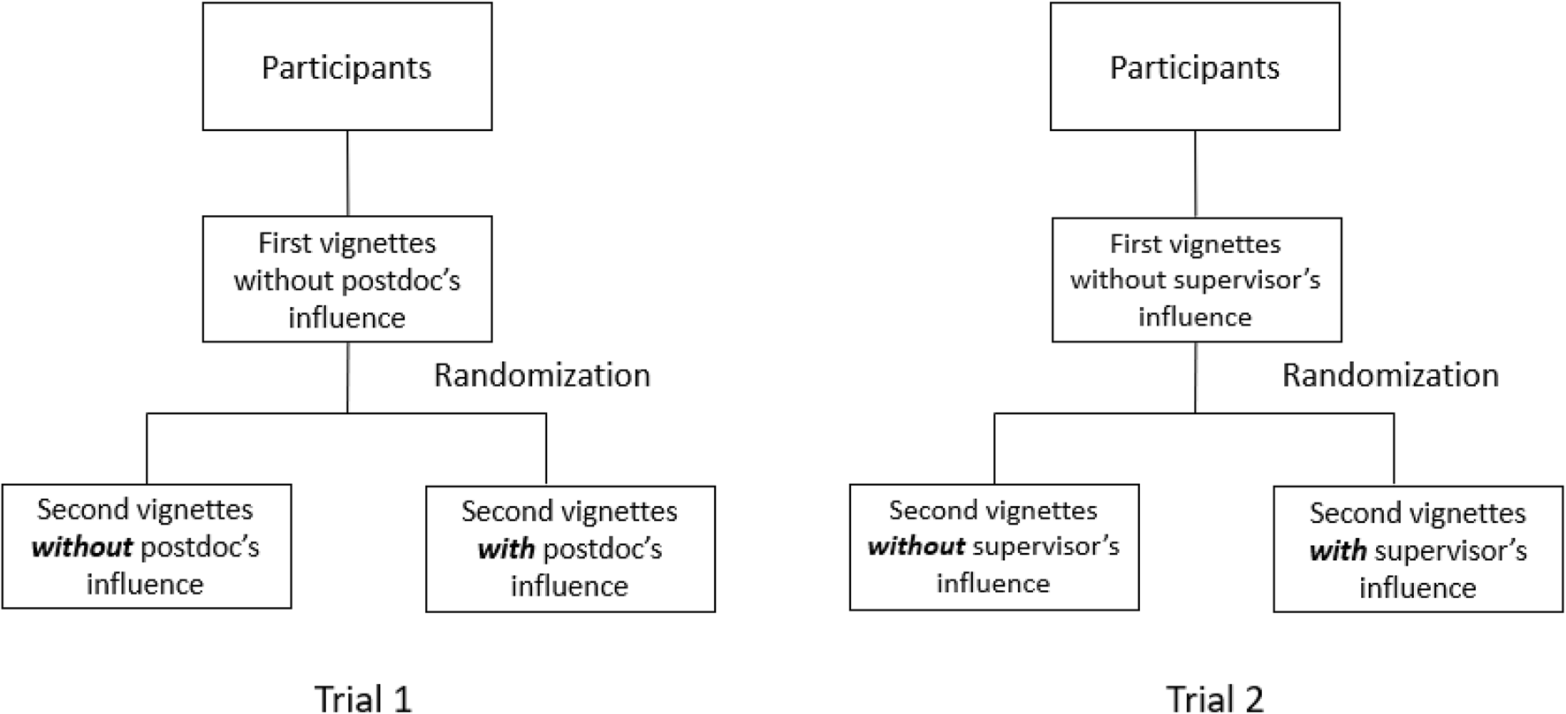
Trial design.

The randomized trials were embedded in an online survey. Each participant answered two vignette-based questions with two different dilemmas in the trial. The first vignette detailed a dilemma *without* environmental factors, aiming to examine the participants’ baseline research practices. For example, should the PhD student add the head of the department as an author of the PhD student manuscript while he/she was not involved in any part of the article (see Table 2)? In the second vignette, participants were randomized to receive the vignette *with or without* an additional sentence describing an environmental factor (i.e. experience of postdoctoral researchers or supervisors’ opinions) that might influence students’ decisions (Fig. 1).

**Table 2:** Example of vignette and the two environmental factors.

Vignette	*The background* Maria is a PhD student in the 2nd year of her doctoral programme *The drama* She has just finished an article related to her PhD project to submit to a journal. She worked with her supervisor to design the study and two PhD colleagues to extract and analyse the data. She led the writing of the manuscript and all co-authors provided comments on earlier drafts and approved the final manuscript *The dilemma* Before submitting the article, Maria is wondering if she could include the Head of the Department as one of the co-authors, although she has never discussed her work with him before. Because he is a well-known expert scientist in the field, adding his name on the list of co-authors will improve the likelihood that her article is accepted in a top-tier journal
First environmental factor: postdoctoral researcher experiencing similar situation and choosing the DRP	Maria discusses with Alex, a postdoc who have been working in the department for several years. Alex confirms that he included the Head of the Department as last author in all of his articles
Second environmental factor: supervisor who does not object the DRP	Maria discusses with her supervisor. Her supervisor tells her that he is fine with whatever she decides

### Participant

To recruit participants, we used the 2019 World University Rankings from the Times Higher Education^22^ and QS Top Universities^23^ ranking to identify the top 10 universities in the European region (as defined by the World Health Organization)^24^. We then searched university websites for administrative contacts related to any graduate programs in the biomedical sciences (e.g. pharmacy, medicine, public health, psychology, genetics, etc.). We also used the personal contacts of researchers within the MiRoR consortium to contact administrators of doctoral programs in their own universities. We sent semi-personalized email invitations to explain the purpose of the study and included the link to the survey (Appendix 2). We also used Twitter to disseminate the survey using the networks of the authors and the MiRoR consortium.

### Environmental factors evaluated in the RCTs

We tested the impact of two environmental factors on PhD students’ research practices in two separate RCTs:

(1) Exposure to a post-doctoral researcher who experienced a similar situation and chose the DRP; (2) exposure to a supervisor who was consulted and did not object to the DRP.

1. The first trial explored the impact of exposure to an environment in which a postdoctoral researcher described the experience of committing a detrimental practice in a similar situation vs*.* no exposure to this environmental factor (no consultation with colleagues).
2. The second trial explored the impact of exposure to an environment where the supervisor was consulted, but the supervisor did not object to the detrimental practice (i.e. the supervisor agreed with the student’s decision) *vs.* no exposure to this environmental factor (no consultation with the supervisor).

An example of a vignette is provided in Table 2 and an additional example can be found in Appendix 3.

### Outcomes

Participants were asked to indicate the solution that they preferred on a semantic differential scale, rated from definitely (5) to maybe (1) a preference for solution A or from maybe (1) to definitely (5) a preference for solution B. The DRPs were randomly reported as solution A or solution B to avoid bias related to the presentation of the scale. We derived a score from − 5 to + 5, where positive scores indicated the choice of DRP and negative scores indicated good practice or no DRP.


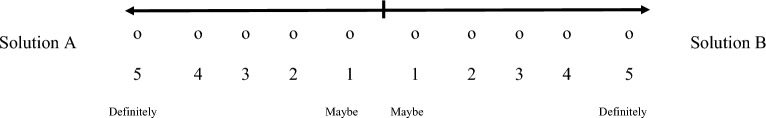


### Sample size calculation

In each randomized trial, participants responded to a first vignette without environmental factors and then, after randomization, a second vignette with or without environmental factors. To assess a mean difference of 1.0 for the outcome between groups on a scale from − 5 to + 5 with a standard deviation of 2.5, for each trial, a sample size of 264 was needed to detect the effect size of 0.4 with a power of 90% and α risk of 5%. Accounting for a 10% attrition rate, we required a sample size of 300 participants (150 in each arm) so that each vignette was evaluated 15 times in each arm.

### Randomization

For each trial, an IT manager generated a random assignment sequence using a block of 10; the list was not disclosed to the investigators. Allocation concealment was ensured using a computerized random assignment system. Randomization was programmed and piloted. Participants who did not complete the survey were replaced by other participants, whereas the intervention and control arms were ensured to be well balanced across vignettes. When the required sample size of the first trial was reached (i.e. 300 participants), subsequent participants were enrolled in the second trial.

### Blinding

It was not possible to blind participants to the intervention. However, the participants were blinded to the study hypothesis. All the participants were informed of the first objective of the study, which was to explore the research practices of PhD students facing dilemmas. However, they were not informed of the second objective of evaluating the impact of environmental factors on their practices.

### Analysis

Statistical analyses were performed using SAS software, version 9.4 (SAS Institute, North Carolina, US) and R 4.4.1.^25^ The analysis population was comprised of participants who completed the trial, i.e. who read two vignettes. The primary outcome was the mean preference score. The difference in means between the groups in each trial was analyzed using an intercept and slope random linear model with a fixed group effect, random vignette effect, and vignette-group interaction effect (assuming an unstructured covariance matrix). Inferences were based on restricted maximum likelihood. Estimated coefficient on fixed group effect represent difference in means and its standard error allow to calculate 95% confidence intervals (CIs). Random effects allowed accounting for within-group clustering (i.e. each vignette was evaluated many times in each arm), between-group clustering (i.e. pairing of vignettes used in the two arms of the trial), and the possibility that scores may vary between vignettes.

The secondary outcome was the proportion of students who chose the DRP (i.e. rated at least 1 on a scale of − 5 to 5 for the DRP). For secondary outcomes, we estimated both the absolute risk difference of proportions of students who chose the DRP and relative risk (with 95% CIs) with a log binomial model under a GEE framework assuming an exchangeable correlation structure in the context of clustered data due to vignettes. P < 0.05 was considered statistically significant.

Furthermore, we conducted a post hoc analysis to examine factors associated with DRP. Vignettes assessed before randomization were pooled from the two trials. The factors considered were determined a priori: gender, research experience, training on research integrity, and pressure to publish. We estimated odds ratios (with 95% CIs) using univariate and multivariate logistic models under a GEE framework, assuming an exchangeable correlation structure in the context of clustered data due to vignettes.

### Data sharing

The dataset will be shared on an open-access platform (https://zenodo.org) after the article has been published. The corresponding author could provide the dataset upon request.

### Ethics approval and consent to participate

This study was approved by the Institutional Review Board of the Inserm (Comité d’Évaluation Éthique, IRB 00003888, Reference Number: 19-587).

## Results

### Participants

**Figure 2:**
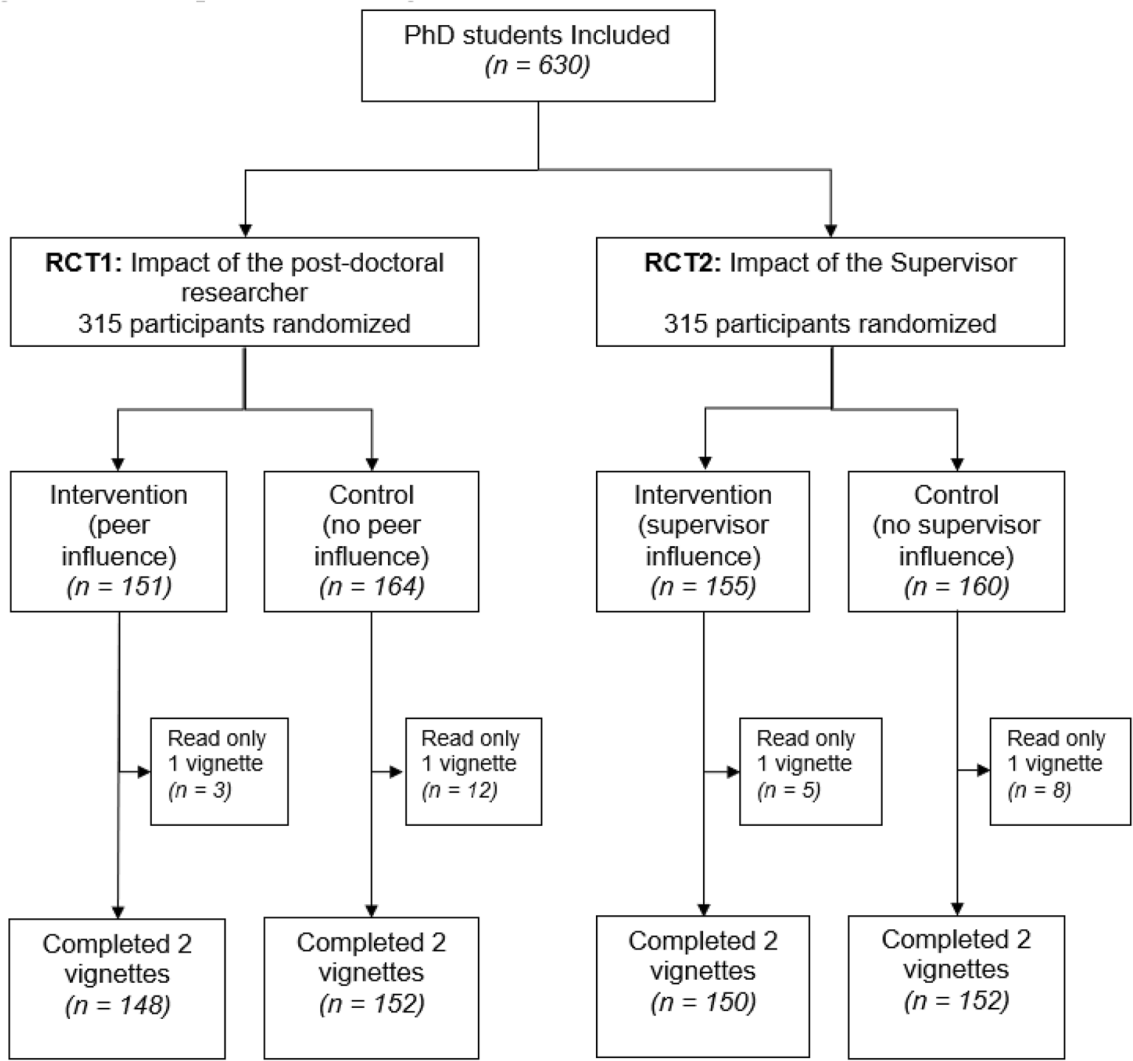
Participants’ flow diagram.

The survey was conducted from January 21 to February 28, 2021. We emailed 179 contacts at 129 universities up to three times each. Fifty-two participants agreed to participate and forwarded our invitation to their students. Sixteen participants declined participation, and 111 were unresponsive after three attempts. Further, 3200 persons opened the link to the survey, and 923 accessed the link from social media. A total of 861 confirmed that they were PhD students and consented to participate. Overall, 630 participants were randomized to assess the impact of the exposure to the postdoctoral researcher (n = 315) and the supervisor (n = 315). Twenty-eight participants did not read the second vignette and were, consequently, excluded from the analysis (15 in the first trial and 13 in the second trial). Participants’ flow diagram is presented in Fig. 2.

### Demographic characteristics

Table 3 summarizes the characteristics of the students who completed the study. Overall, 58% of them were female students. They belonged to 43 different countries (Appendix 4), and most participants (92%) responded in English. The median [IQR] years of enrollment in their PhDs was 2.0 [1.0–3.0]. Most students (80%) were enrolled in full-time PhD programs. Two-thirds indicated that they had some experience in research prior to enrolling in the PhD program. Overall, 61% received training in research integrity, and 65% of them indicated that they received training only once during the entire PhD program. Nearly half of the students reported experiencing a great deal of pressure to publish in their academic institutions.

**Table 3:** Participant characteristics.

Characteristics (*N* = *602)*	N (%), N = 602
Location (i.e. five countries with the highest number of participants)	
France	88 (14.6%)
Germany	55 (9.1%)
Italy	44 (7.3%)
Belgium	45 (7.5%)
Finland	41 (6.8%)
Sex	
Male	182 (30.2%)
Female	348 (57.8%)
Other	7 (1.2%)
Missing data	65 (10.8%)
Research experience prior to enrolling in a PhD program	
Yes	409 (67.9%)
Missing data	69 (11.5%)
Supervision	
I can always discuss with my supervisor and I can make final decision	324 (53.8%)
I can sometimes discuss with my supervisor and I can sometimes make the decision	157 (26.1%)
I always have to follow decision of my supervisor	39 (6.5%)
Missing data	82 (13.6%)
Pressure to publish	
Not much/little	82 (13.6%)
Somewhat	169 (28.1%)
Much/a great deal	282 (46.8%)
Missing data	69 (11.5%)
Training	
Receiving training on research integrity	369 (61.3%)
Missing data	65 (10.8%)
Frequency of research integrity training in your institution *(n* = *369)*	
Once a year	67 (18.1%)
Once every 2 years	12 (3.3%)
Only once during PhD training	240 (65.0%)
Missing data	50 (13.6%)

### Research practice of PhD students

**Figure 3:**
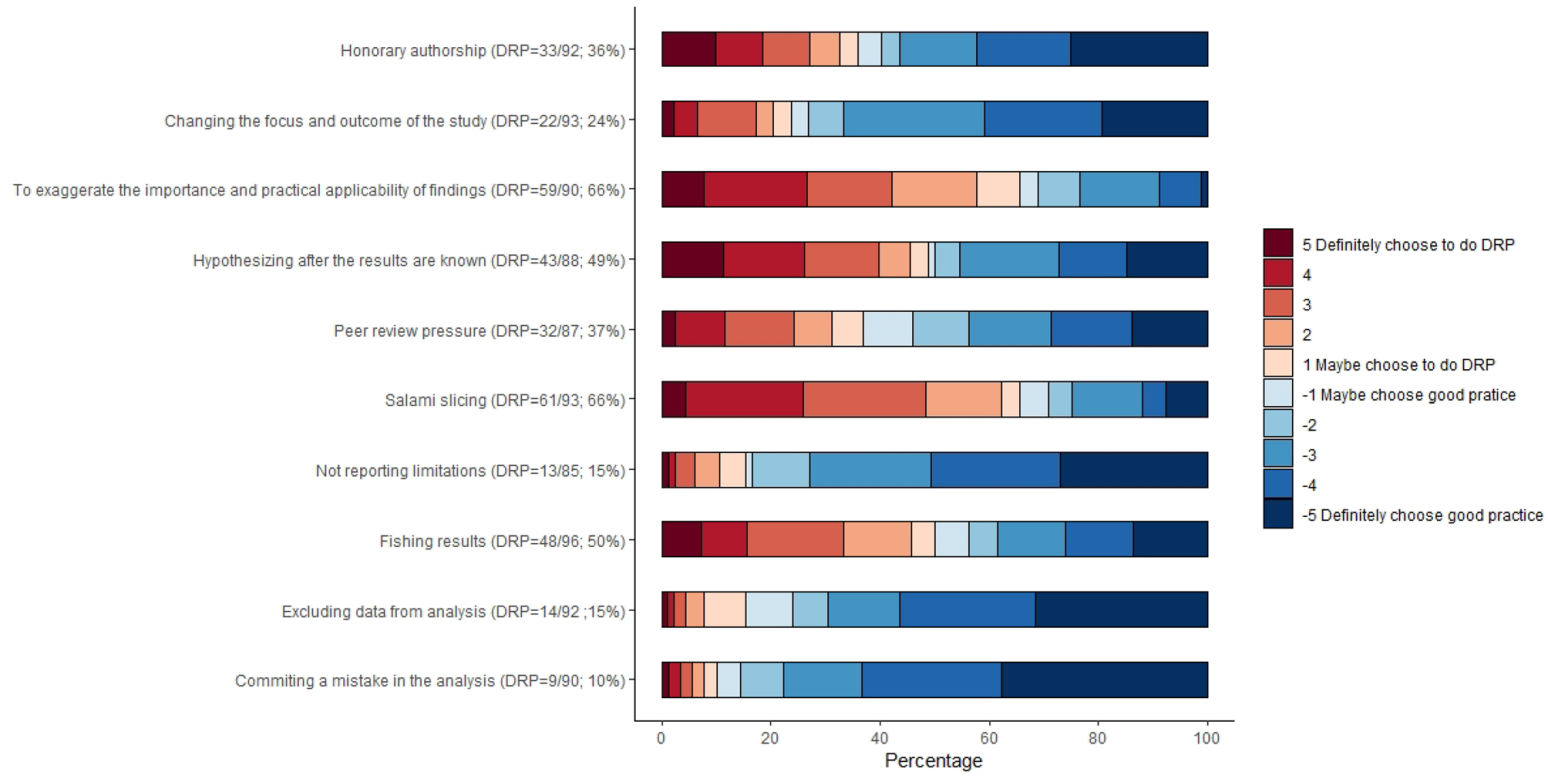
PhD students’ research practice assessed on first vignette (before intervention).

A total of 602 PhD students completed the first vignette (without any environmental factors) and provided baseline information on their research practices. Of these, 221 (37%) selected DRPs while answering the vignettes. Figure 3 presents the distribution of the students’ choices across the vignettes. The proportion of PhD students selecting DRPs varied substantially across vignettes. Overall, 66% of the students chose to split their research into several publications (i.e. salami slicing), 66% chose to exaggerate the importance of findings, half of the participants chose to run multiple analyses and report only results with statistical significance (i.e. fishing results), and half decided to modify the hypothesis after seeing the results (i.e. harking). In contrast, 10% chose not to report a mistake in the analysis to the editor; 15% chose to exclude data from analysis to improve results and 15% chose not to report their study limitations.

### Impact of exposure to a postdoctoral researcher’s experience

**Figure 4:**
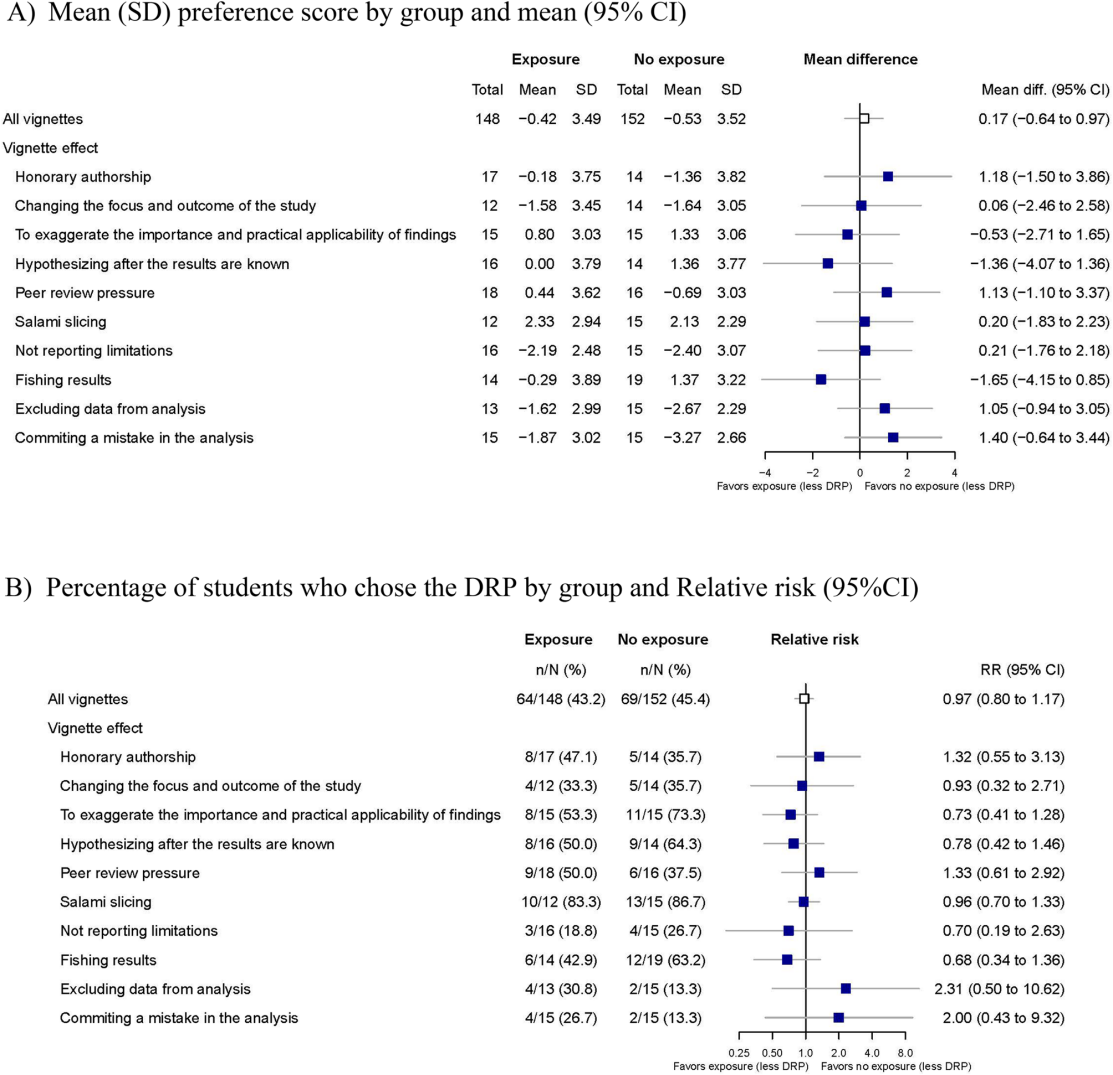
Impact of the exposure to a postdoctoral researcher’s experience environmental factors on PhD student’s research practices.

Of 315 randomized students, 302 (148 intervention vs. 152 control) completed the trial. Exposure to the postdoctoral researcher did not have a significant impact on PhD students’ choice of DRP across vignettes. The mean (SD) preference score for the vignettes with or without postdoc influence was − 0.42 (3.49) versus − 0.53 (3.52) (mean difference [95% CI] 0.17 [− 0.65 to 0.99;], p = 0.65) (Fig. 4A). The proportion of students choosing DRP was 43.2% vs 45.4%; (relative risk (RR) 0.97 [0.80; 1.17]; risk difference (RD) − 1.6% [− 11.9; 8.8]) (Fig. 4B).

### Impact of the supervisor

**Figure 5:**
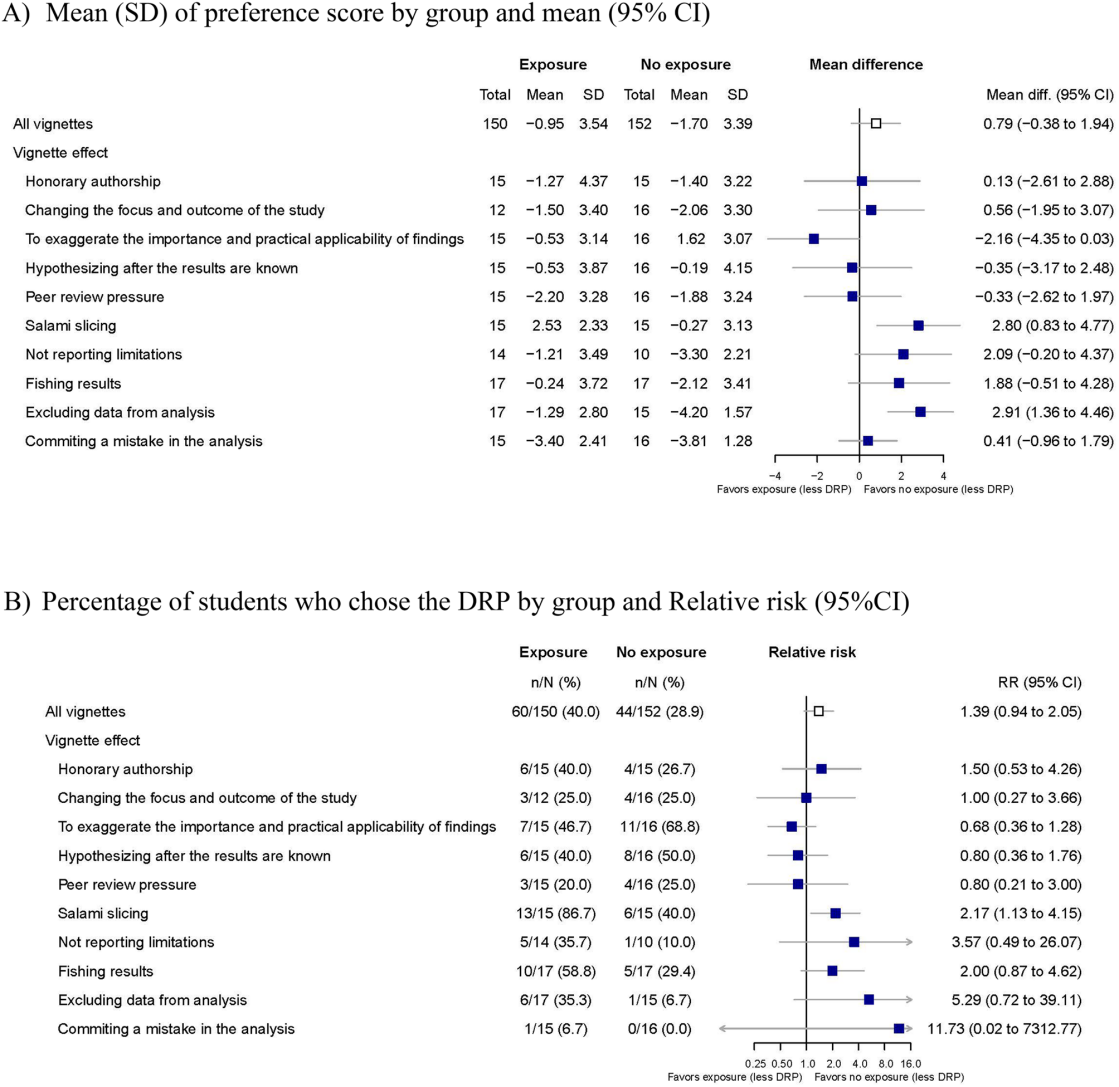
Impact of the exposure to a supervisor who does not object DRP on PhD student’s research practices.

Of the 315 randomized students, 302 completed the trial. Of these, 150 students were exposed to a supervisor who did not oppose the DRP, and 152 students were in the control group. The supervisor did not have an impact on the preference score between DRPs and no DPRs. The mean (SD) score for vignettes with versus without exposure to the supervisor was − 0.95 (3.54) versus − 1.7 (3.39) (mean difference [95% CI] 0.79 [− 0.38; 1.94], p = 0.16) (Figs. 5A). The proportion of students choosing the DRP was 40.0% vs. 29.0% (relative risk (RR) 1.39 [0.94; 2.05]; risk difference (RD) 11.2% − 0.4; 26.3]) (Fig. 5B).

### Post-hoc analysis

The post-hoc analysis explored the association between gender, research experience, training, pressure to publish, and the choice of DRP, but it did not show any statistically significant results (Table 4).

**Table 4:** Logistic regression analysis of factors affecting detrimental research practices.

	Univariate analysis*		Multivariate analysis**		p
	OR	[95% CI]	OR	[95% CI]	
Female vs male	1.28	[0.86, 1.89]	1.26	[0.87, 1.84]	0.22
Research experience	0.78	[0.58, 1.05]	0.81	[0.57, 1.14]	0.23
Training on research integrity	1.05	[0.64, 1.73]	1.00	[0.60, 1.66]	0.99
Pressure to publish					
Somewhat vs not much/little	1.25	[0.88, 1.77]	1.27	[0.88, 1.85]	0.20
Much/A great deal vs not much/little	1.03	[0.75, 1.41]	1.01	[0.75, 1.34]	0.97

*n = 530, n = 533, n = 537, n = 533, for univariate analysis, respectively.

**n = 523 for multivariate analysis.

## Discussion

This study provides insights into the research practices of biomedical doctoral students in Europe, performance pressures (i.e. the necessity to publish). Our results showed that students frequently chose DRP, and the DRP depended considerably on the dilemma they faced. The most frequently chosen DRPs were exaggerating the importance of the finding (i.e. adding spin), salami slicing, and fishing results, indicating potential situations that may need more attention. Our study also indicated that nearly half of PhD students were under considerable pressure to publish. This pressure might impact the research practices of PhD students, leading them to commit questionable practices to fulfill quantitative requirements from research institutions^26^. This situation was recently reported in a survey of Taiwanese doctoral students who committed questionable authorship practices due to the pressure to achieve academic success^27^.

To our knowledge, this is the first study to explore the impact of environmental factors (i.e. the roles of post-doctoral researchers and supervisors) on research practices when facing a dilemma that could lead to DRPs with a large cohort of European PhD students in the biomedical sciences. On an average, we did not find evidence of an impact of postdoctoral researchers and supervisors on student practices. However, the results vary considerably across vignettes and this study lacks enough power to perform an analysis at the vignette level. Further studies are needed to explore the impact of the environment on the dilemma most frequently responsible for DRPs. Situations in which PhD students need more decision-making support and potential interventions to prevent DRPs need to be identified.

Our study had some limitations. First, our response rate from university administrators was not ideal, and several declining participation remarked that their students had “survey fatigue” from COVID-19 related online surveys circulating during our recruitment period. We initially delayed our recruitment period in consideration of this; however, this general fatigue may have affected our survey engagement and completion rates. Second, we focused on biomedical doctoral students in Europe and geared recruitment efforts geared towards these participants. Therefore, the generalizability of our findings is skewed towards a European context, where there may have been more initiatives to raise awareness of research integrity issues^28–30^ than in other settings^31^. Additionally, decisions made in a survey might not reflect their actual practices. Nevertheless, we designed a vignette to closely represent situations that PhD students may face in their practice. We conducted a literature review and a qualitative study to support the development of vignettes. This process allowed us to prioritize the dilemmas that were most relevant to the context of PhD students and to express the vignettes in a way that participants could easily relate to. Nevertheless, our study does not address institutional policies within which students are working and institutional responsibilities for how students are practicing science. Finally, while we made the survey available in several European languages, most respondents chose to complete the survey in English, which is not their native language.

Our results clearly probe into the evaluation of academic performance at research institutions. Changes in the methods of evaluating PhD students’ academic performance are urgently needed to incentivize good research practices. Some institutions have employed new methods to assess researchers, focusing on research quality and contribution to other colleagues’ work and career, rather than on quantitative metrics^32^. Training in research integrity should be revised. Doctoral students are the next generation of researchers and will represent the field in the future. Therefore, PhD students should be trained to be aware of DRPs, what to do if encountered, and how to enact measures against these detrimental practices. Research integrity training should be commenced at the early stages of career development, including at bachelor’s and master’s levels and should be provided on a regular basis to update rules and guidelines and maintain high attention to issues.

## Conclusion

Biomedical doctoral students frequently chose DRP when facing dilemmas. We did not find any evidence of the impact of post-doctoral researchers and supervisors. However, there was some variability across the vignettes, and more studies are needed. This study also provides insights into the research practices of PhD students in biomedical disciplines across multiple countries. The study findings can be useful to universities, research integrity bodies, research funders, and training providers. These stakeholders should provide training to students and staff as well as supervisors and post-doctoral researchers.

### Supplementary Information

Supplementary Information.
