# Supplementary material for: Biomedical doctoral students’ research practices when facing dilemmas: two vignette-based randomized control trials
Source: Sci Rep. 2023 Sep 29;13:16371. doi: 10.1038/s41598-023-42121-1 (PMC10541422; doi:10.1038/s41598-023-42121-1)
Supplement: Supplementary file 1 — Supplementary Information. [file 41598_2023_42121_MOESM1_ESM.pdf]

Environmental influences on biomedical doctoral students' research practices when facing dilemmas: vignette-based randomized control trials

Nguyen, V.[1,2], Sharp, M.K.[1,3,4], Superchi, C.[1,5], Baron, G.[1,7], Glonti, K.[1,3], Blanco, D.[1,6], Olsen, M.[1,8], Vo Tat, T.[1,9], Olarte Parra, C.[1,10], Névéol A.[11], Hren, D.[3],

Ravaud P.[1,7], Boutron I.[1,7,*]

Appendix 1. Semi-Structured Interview Guide

Introduction

My name is […], I am from […]. Currently I am a PhD student (at […] University/in the field of […]). I am working on a project that focuses on what early researchers think about questionable research practices. I am interested in your understanding of this issue and your personal experience with it.

During the interview, please feel free to share as much as you would like. With your permission we will be recording and transcribing the interview. All identifying information such as your name and institutions will be redacted to ensure your anonymity. If you would like, you can request a copy of your transcript.

**Background**<br>*Aim:* to elicit information from the interviewee regarding their academic background, past research experience and current research studies. Within current research studies we would like to learn more about their role.	1) To start off with, can you tell me about your educational background and experience with research, both what you did before and what you are doing currently?<br>• prompt: chronological order, current research position
**Defining the Issue**<br>*Aim*: to find out about interviewee's understanding of QRP, the meaning they attribute to it and general examples they consider to represent QRP.	2) As you know, in this project we are interested in graduate students' experiences with "questionable research practices". What comes to mind when you hear that phrase? (What does it mean to you?)<br>• probe: Can you specify research practices you consider to be bad or questionable (apart from obvious misconduct such as falsification, fabrication, plagiarism)?<br>• probe: What do you consider to be the most serious questionable research practices?<br><br>3) How has your training so far addressed these issues?_<br>• probe: Can you tell me of a situation where the training you received helped you make decisions?<br>**no training** has addressed<br>• probe: How did you learn about this issues?<br>• probe: Whom would you ask for help or advice?

**Personal Experience**<br>*Aim:* to ascertain how the the interviewee feels about the current research climate in light of their own research and training experiences and to probe whether or not there is room for potential QRP's in their own research	4) Lately, one can hear or read about the challenging research climate that we are faced with. People cite things such publication records, grants/funding, reporting bias by journals etc. as current conditions that may potentially effect research practices. How does the research climate that you currently work in (both broad, within the discipline, and local, at the institution or workplace) influence the likelihood of questionable research practices happening?<br>• prompt: give examples of QRP (pressure from peers, supervisors, institutions, funders to publish, to include people in authorship, to use certain methodology/ inappropriate research design, to remove outliers, to omit negative outcomes, to leave out relevant controls)<br>• probe: What measures could you take to prevent negative effects of climate (if any)?<br>• probe: Are there people or resources who can provide guidance and assistance to you? (Either in preventing QRP's or correcting for them if they take place?)<br><br>5) Could you tell me of situation (at your current or any of your previous positions) where you have witnessed or found yourself involved in research practice that you considered questionable?<br>• prompt: Can you tell me a time when you weren't sure what to do?<br>• probe: Please describe/explain what happened?<br>• probe: How did you act in those situations?
**View of the Broader Issue**<br>*Aim:* to find out about interviewee's perspective about the causes and frequency for QRP	6) In your experience, when are these practices most likely to occur in your research field?<br>• probe: Who are the researchers that are most vulnerable to QRP?<br>• probe: How common are such practices?<br><br>7) Do you see any ways of solving some of the problems you mentioned?<br>• probe: What advice would you have for other students in relation to QRPs?<br>• probe: And advice to supervisors/ mentors, journal editors etc.?
**Conclusion:**<br>*Aim:* to provide the interviewee with the opportunity to sum up their thoughts, offer any advices and/ or final words<br>This is also an opportunity for the interviewer to clarify any aspects there was uncertainty over.	Interviewer sums up the points discussed and asks for clarification if needed.<br><br>8) Is there anything else you would like to add?<br>• prompt: anything we haven't covered that might be important

Appendix 2. Recruitment Letters

<u>Participation Information Sheet for Qualitative Interviews for Case Vignette Development</u>

PARTICIPANT INFORMATION SHEET

Questionable research practices – qualitative interview

Researcher introduction

We are PhD students in the European Joint Doctorate program funded by Marie Skłodowska-Curie Actions, dedicated to Method in Research on Research (MiRoR). The project aims to reduce research waste and increase research value.

Project description and invitation

Research suggests that questionable research practices are prevalent in medical research and may have a deteriorating effect on the quality as well as credibility of research. Existing studies in this field mostly focused on experiences of established researchers. Less research has been conducted on early career researchers such as PhD students.

For this reason, we are undertaking research in order to gain understanding of PhD students' perception and experience on questionable research practice. We would like to invite you to take part in our research! This would involve a qualitative interview where we will ask about your understanding towards questionable research practices and your experience of encountering these issues. The interview will take about 45 minutes.

All PhD students in biomedical disciplines registered in any University in European Union are eligible for this research. The interview would, with your consent, be audio tape-recorded, and will be transcribed by PhD students of MiRoR. Participation in this research is entirely voluntary, and you would be able to withdraw from the research, without giving reasons, prior to, and during, the interview. All information you provide in an interview is confidential. Identifying information related to your identity, your research project and your institution will be changed, and a pseudonym given to any of your data used in publications arising from this research. Your interview will also be used to inform the development of a survey with the same topic which will be sent to a larger sample of PhD students in biomedicine.

The study has received ethical approval from INSERM ethics committee with reference number 17-387. Data collected will be saved to a computer file accessible by the INSERM METHODS team. In keeping with the "Informatique et libertés" law, you can assert your right to access data which concerns you and have it rectified by notifying Professor Isabelle Boutron at isabelle.boutron@aphp.fr

If you have any question about this research, please contact Professor Isabelle Boutron at the same email address above.

Recruitment Emails to University Representatives

Initial Invitation

Dear [TITLE FIRST NAME LAST NAME],

We are contacting you as [POSITION] at [UNIVERSITY]. We are from an international research and training programme called Methods in Research on Research (MiRoR), funded by the European Union's Horizon 2020 programme. Our research network aims to explore and improve biomedical research methods.

We are currently conducting a survey, which aims to understand what decisions PhD students make when facing difficult situations during their research. For example, what actions are taken in situations with publication pressure or having unexpected negative findings.

As it is challenging to reach individual PhD students directly, we are contacting you to kindly ask if you would help us and distribute the survey to the biomedical PhD students at your faculty/university? We have attached a pdf copy of the survey. If you agree to invite your students to participate, we will send you a live hyperlink.

Please let us know if you would be willing to forward the survey or if you have any questions.

Thank you very much for your time and consideration. Your contribution and reply is greatly appreciated.

Kind regards,
Camila Olarte, Melissa Sharp, and Van Thu Nguyen
on behalf of the MiRoR Team

Centre of Research in Epidemiology and Statistics
Université de Paris, CRESS, INSERM, INRA
F-75004 Paris, France
essence@clinicalepidemio.fr
MiRoR project
This project has received ethical approval from the University of Paris Descartes.

Follow-up Email
Dear [TITLE FIRST NAME LAST NAME],

Thank you for taking the time to consider our proposal. We are grateful that you have agreed to distribute the survey among your students. We have added a copy of the survey invitation below. Please feel free to personalise it as you see fit and share the survey link (URL) using any relevant social media accounts (e.g., Twitter) you may have. Again many thanks for your help!

Best,
Melissa

Dear PhD student,

Have you ever had to make a difficult decision in your research?

We would like to invite you to participate in a quick survey, which contains interesting stories of early stage researchers, just like you, who have to make a decision related to their research. Each story presents a tricky situation where there is no right or wrong answer.

The survey can be accessed in the following link: URL. By completing this 15 minute survey, you will help us develop training materials to support PhD students in decision making in such difficult situations.

We look forward to receiving your reply and don't hesitate to forward the survey with your PhD colleagues in the biomedical sciences - thank you very much!

Kind regards,
Camila Olarte, Melissa Sharp and Van Thu Nguyen
on behalf of the MiRoR Project

Centre of Research in Epidemiology and Statistics
Université de Paris, CRESS, INSERM, INRA
F-75004 Paris, France
MiRoR project
This project has received ethical approval from the University of Paris Descartes.

Appendix 3. Additional example of vignette and the two environmental factors:

A. COMMITING A MISTAKE IN THE ANALYSIS

The background

Mike, a PhD student, conducted an experiment to examine if a compound X could reduce tumour size in mice.

The drama

He analysed the data which showed that the use of compound X was significantly associated with reduction in tumour size. He wrote the paper and it was published very quickly in a peer-reviewed journal. One month after the paper was published, he realised he made a mistake in the analysis. After he corrects the analysis, the results show that the observed association of X with tumour size in mice is weaker than in the initial analysis, and no longer statistically significant.

The dilemma

With this mistake, the editor might request to withdraw the paper which might affect his PhD, and Mike wonders if he should report the mistake.

Research climate	Mike talks to Julie, a postdoc who has been working in the lab for several years. Julie says that Mike doesn't need to do anything. If the editor notices the problem, he will inform Mike later.
Role of supervisor	Mike talks to his supervisor. His supervisor says that she is fine with whatever Mike decides.

B. TO EXAGGERATE THE IMPORTANCE AND PRACTICAL APPLICABILITY OF FINDINGS

The background

Peter has just completed the first manuscript for his PhD project in which he conducted an experiment on animals.

The drama

He shows his manuscript to another researcher from his institution, who suggests him to change the abstract conclusion to highlight that these results could have a huge impact on public health.

The dilemma

Peter wonders if he should make this optimistic statement in the abstract conclusion in order to make the results more palatable and interesting for the audience.

Research climate	Peter talks to Daniel, a postdoc who has been working in the same lab for several years. Daniel agrees with the researcher and says he often writes strong and positive statements in the abstract.
Role of supervisor	Peter talks to his supervisor. His supervisor says that he is fine with whatever Peter decides.

Appendix 4. List of countries

**Location** (in descending order)	**N (%)**<br>**N=602**
France	88 (14.6%)
Germany	55 (9.1%)
Italy	44 (7.3%)
Belgium	45 (7.5%)
Finland	41 (6.8%)
Spain	26 (4.3%)
United Kingdom	26 (4.3%)
Hungary	24 (4.0%)
Netherlands	21 (3.5%)
Ireland	20 (3.3%)
Norway	17 (2.8%)
Sweden	17 (2.8%)
Switzerland	16 (2.7%)
Greece	14 (2.3%)
Romania	13 (2.2%)
Lithuania	11 (1.8%)
Croatia	7 (1.2%)
Georgia	7 (1.2%)

**Location** (in descending order)	**N (%)** **N=602**
Portugal	4 (0.7%)
United States	4 (0.7%)
Brazil	3 (0.5%)
Canada	3 (0.5%)
Estonia	3 (0.5%)
Iceland	3 (0.5%)
Australia	1 (0.2%)
Austria	1 (0.2%)
Bosnia and Herzegovina	1 (0.2%)
China	1 (0.2%)
Colombia	1 (0.2%)
Curaçao	1 (0.2%)
Denmark	1 (0.2%)
Egypt	1 (0.2%)
Ethiopia	1 (0.2%)
India	1 (0.2%)
Kenya	1 (0.2%)
Paraguay	1 (0.2%)
Poland	1 (0.2%)
Saudi Arabia	1 (0.2%)

**Location** (in descending order)	**N (%)**<br>**N=602**
Serbia	1 (0.2%)
Singapore	1 (0.2%)
Tunisia	1 (0.2%)
Turkey	1 (0.2%)
Viet Nam	1 (0.2%)
Unknown location	71 (11.8%)

Percentage may not sum to 100 due to rounding.
